# Supplementary material for: The Kaposi’s sarcoma-associated herpesvirus viral genome packaging accessory factor ORF68 forms cytoplasmic puncta dependent on the viral tyrosine kinase
Source: bioRxiv. 2026 Feb 23:2026.02.23.707506. Preprint. [Version 1] doi: 10.64898/2026.02.23.707506 (PMC13054539; doi:10.64898/2026.02.23.707506)
Supplement: Supplement 2 [file NIHPP2026.02.23.707506v1-supplement-2.pdf]

bioRxiv preprint doi: https://doi.org/10.64898/2026.02.23.707506; this version posted February 23, 2026. The copyright holder for this preprint (which was not certified by peer review) is the author/funder, who has granted bioRxiv a license to display the preprint in perpetuity. It is made available under aCC-BY 4.0 International license.

SUPPLEMENTARY FIGURES

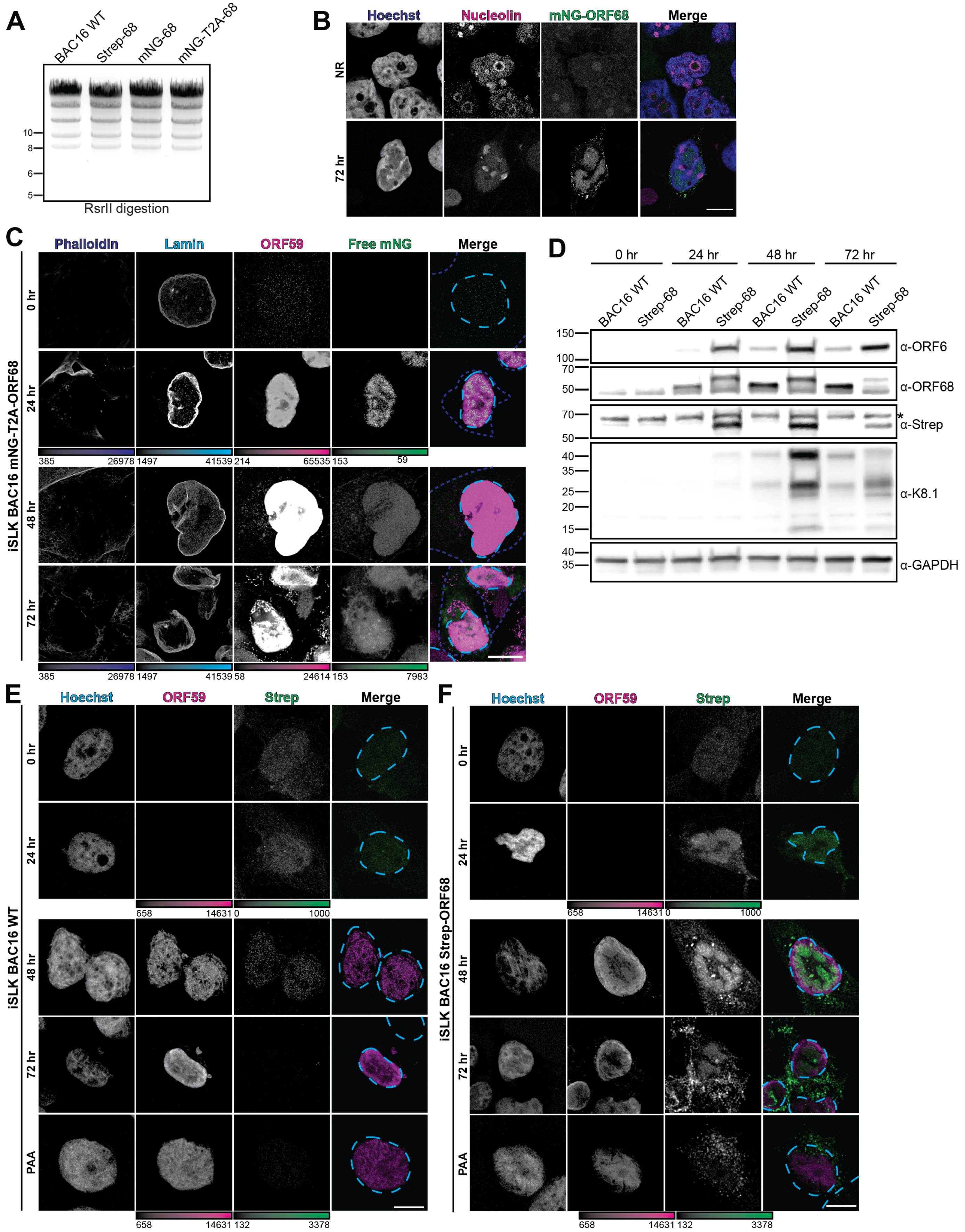

SUPPLEMENTARY FIGURE S1. Alternate tagging of ORF68 produces a similar punctate phenotype unrelated to mNG expression. A. Recombinant TSP-ORF68, mNG-ORF68, and mNG-T2A-ORF68 BACs were digested with RsrII to assess that no large-scale rearrangements occurred during cloning. **B.** Z-slices of iSLK mNG-ORF68 cells at 72 hours post-

bioRxiv preprint doi: https://doi.org/10.64898/2026.02.23.707506; this version posted February 23, 2026. The copyright holder for this preprint (which was not certified by peer review) is the author/funder, who has granted bioRxiv a license to display the preprint in perpetuity. It is made available under aCC-BY 4.0 International license.

reactivation, stained for Hoechst (blue) and nucleolin (magenta), representative of at least 5 cells from 2 biological replicates. **C.** Z-slices of iSLK cells expressing mNG-T2A (green) separately from ORF68 at 0, 24, 48, and 72 hours post reactivation. Stained for phalloidin (blue dashes), Lamin A/C (cyan dashes), and ORF59 (magenta), representative of at least 5 cells from 2 biological replicates. **D.** Western blot of whole cell lysate (25 µg) from iSLK WT or TSP-ORF68 cells at 0, 24, 48, or 72 hours post-reactivation. We blotted for levels of ORF68, early genes (ORF6), and late genes (K8.1, ORF26). GAPDH is the loading control. Asterisk (*) indicates a nonspecific band. 3 biological replicates. **E.** Z-slices of lytic iSLKs expressing WT ORF68 at 0, 24, 48, and 72 hours post reactivation, with or without PAA treatment. Stained for Hoechst (cyan dashed line), Strep (green), and ORF59 (magenta), representative of at least 5 cells from 1 biological replicate. **F.** Z-slices of lytic iSLK TSP-ORF68 cells at 0, 24, 48, and 72 hours post reactivation, with or without PAA treatment. Cells were stained for Hoechst (cyan dashed line), Strep (green), and ORF59 (magenta), representative of at least 5 cells from 1 biological replicate. Scale bars are 10 µm; color bars represent the modified minimum and maximum pixel values.

bioRxiv preprint doi: https://doi.org/10.64898/2026.02.23.707506; this version posted February 23, 2026. The copyright holder for this preprint (which was not certified by peer review) is the author/funder, who has granted bioRxiv a license to display the preprint in perpetuity. It is made available under aCC-BY 4.0 International license.

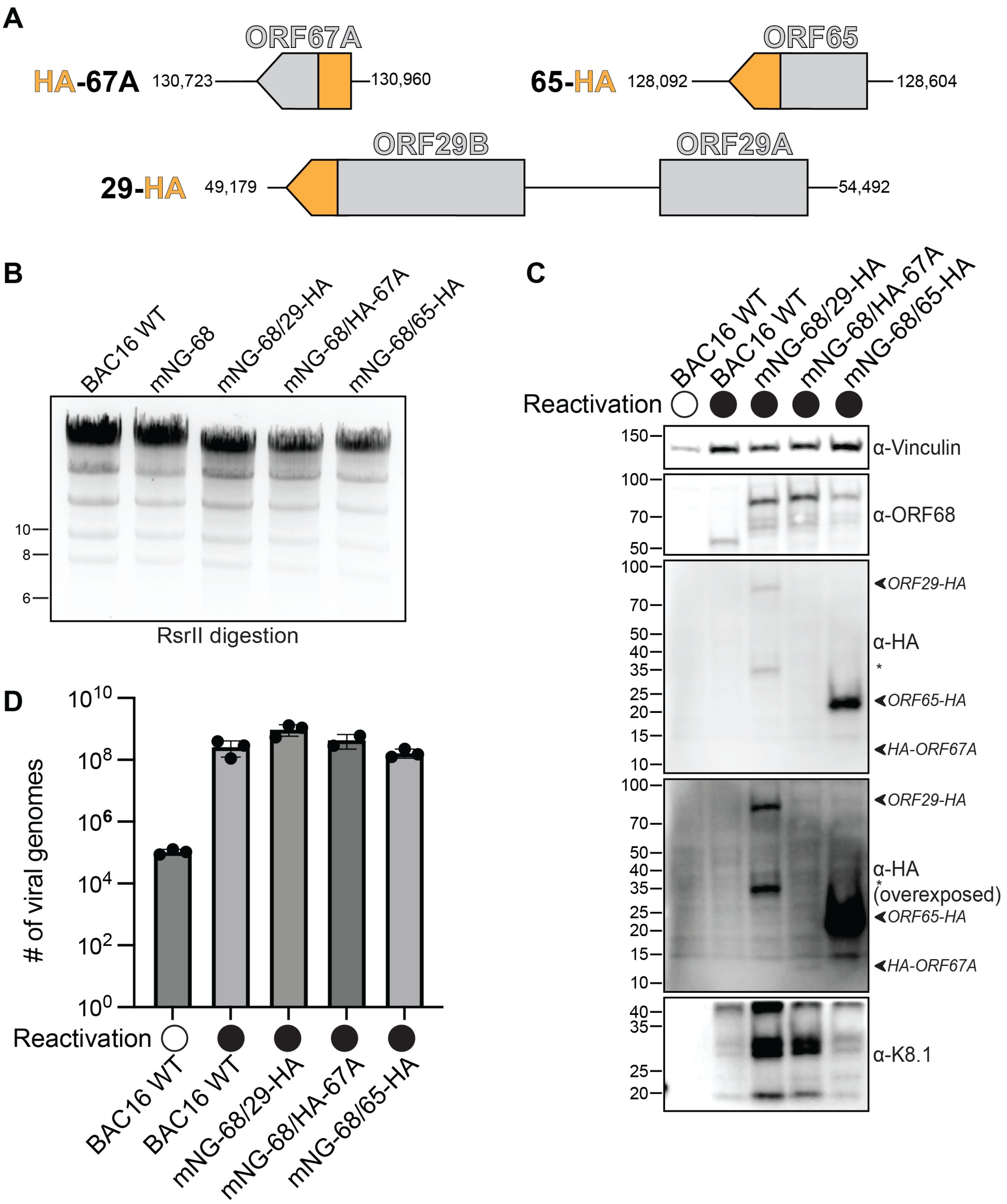

SUPPLEMENTAL FIGURE S2. Tagging ORF68 and capsid or packaging proteins simultaneously does not disrupt the lytic cycle. A. Schematic of the loci in the BAC16 WT genome and insertion of HA-tag at the N-terminus of ORF67 (HA-67), C-terminus of ORF65 (65-HA), and C-terminus of ORF29 (29-HA). **B.** Recombinant mNG-ORF68, mNG-ORF68/ORF29-HA, mNG-ORF68/HA-ORF67A, and mNG-ORF68/ORF65-HA BACs were digested with RsrII to assess that no large-scale rearrangements occurred during cloning. **C.** Western blot of whole cell lysate from reactivated iSLK cells indicate that HA-tagged ORFs (labeled with arrows) are detectable by western blot and tagging does not influence expression of representative early (ORF68) or late (K8.1) genes, representative of three biological replicates. The asterisk refers to an isoform of ORF29-HA. Vinculin is the loading control. **D.** Extracellular virion production of unreactivated or reactivated iSLK cells was

bioRxiv preprint doi: https://doi.org/10.64898/2026.02.23.707506; this version posted February 23, 2026. The copyright holder for this preprint (which was not certified by peer review) is the author/funder, who has granted bioRxiv a license to display the preprint in perpetuity. It is made available under aCC-BY 4.0 International license.

quantified by qPCR from three biological replicates. Filled circles indicate the number of viral genome copies measured for each biological replicate, with the bar representing the mean and error bars depicting the SD.

bioRxiv preprint doi: https://doi.org/10.64898/2026.02.23.707506; this version posted February 23, 2026. The copyright holder for this preprint (which was not certified by peer review) is the author/funder, who has granted bioRxiv a license to display the preprint in perpetuity. It is made available under aCC-BY 4.0 International license.

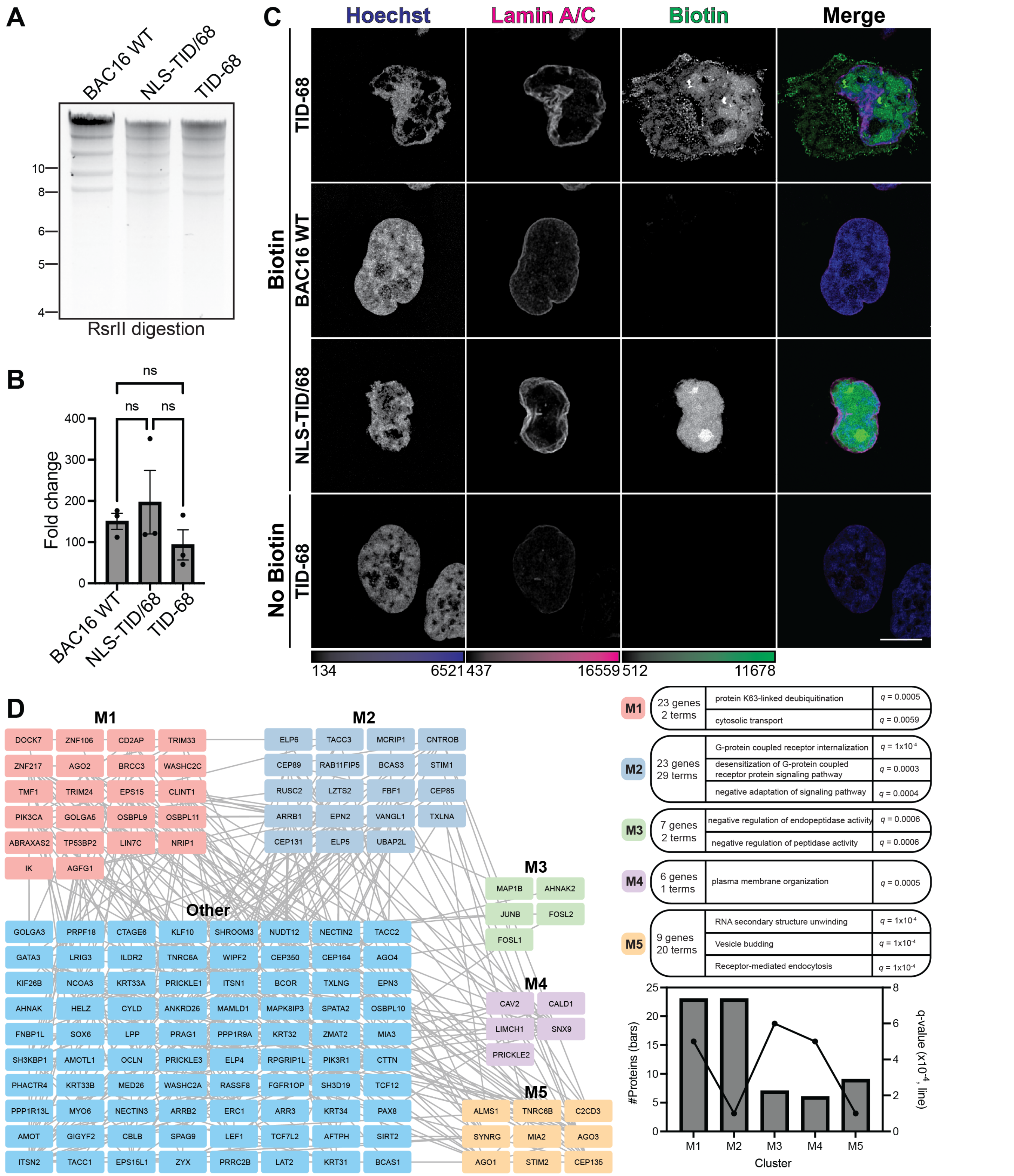

SUPPLEMENTARY FIGURE S3 A. RsrII BAC digestion of BAC16 WT compared to engineered BACS containing NLS-TID/68 and TID-68. **B.** qPCR to quantify viral genome replication at 72 hours post-reactivation of iSLKs infected with BACs WT, NLS-TID/68, or TID-68. Genome replication is not significantly (ns) different between the viruses by RM one-way ANOVA ($p>0.05$). 3 biological replicates. **C.** Z-slices of lytic iSLKs expressing ORF68 WT (BAC16 WT), TurboID-ORF68 (TID-68), or TurboID-T2A-68 (NLS-TID/68) at 48 hours post reactivation, fixed after 15-minute biotin ("Biotin") or DMSO ("No Biotin") treatment. Stained for Hoechst

bioRxiv preprint doi: https://doi.org/10.64898/2026.02.23.707506; this version posted February 23, 2026. The copyright holder for this preprint (which was not certified by peer review) is the author/funder, who has granted bioRxiv a license to display the preprint in perpetuity. It is made available under aCC-BY 4.0 International license.

(blue) and Lamin A/C (magenta). Streptavidin-conjugated to a fluorophore demarcates biotinylated proteins (green). Representative of at least 5 cells from one biological replicate. Scale bar is 10 um. **D.** Proximity-based interaction network of ORF68-specific host proteins. GO term clusters were generated with HumanBase using functional module detection[97]. Bar and line plot indicates the number of proteins and q-values associated with each cluster respectively.

bioRxiv preprint doi: https://doi.org/10.64898/2026.02.23.707506; this version posted February 23, 2026. The copyright holder for this preprint (which was not certified by peer review) is the author/funder, who has granted bioRxiv a license to display the preprint in perpetuity. It is made available under aCC-BY 4.0 International license.

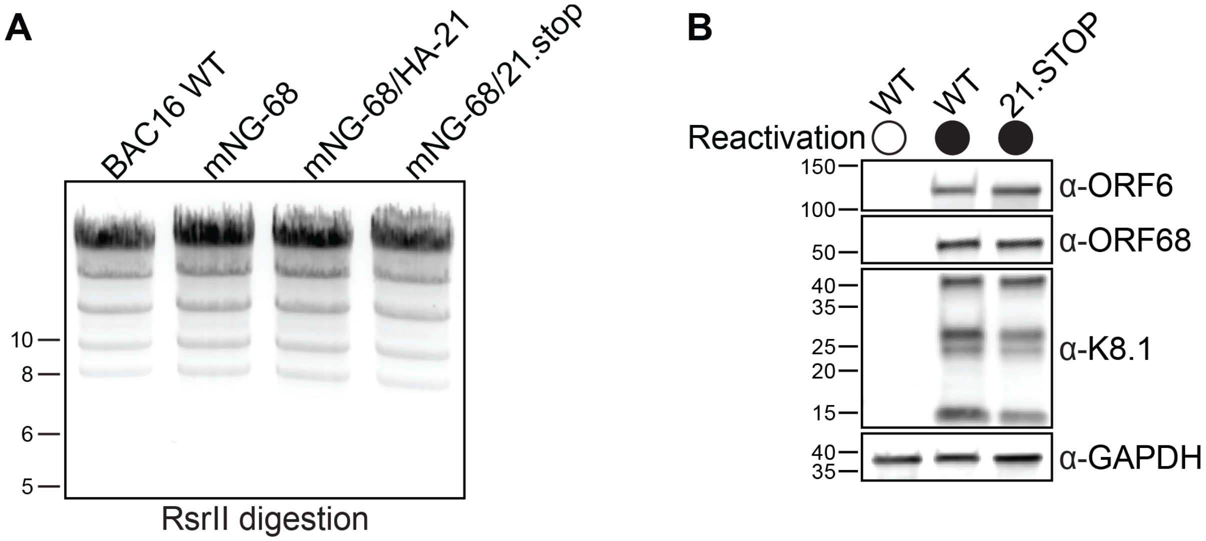

SUPPLEMENTARY FIGURE 4. Generation of KSHV cell lines harboring ORF21-HA and ORF21.stop mutations. A. Recombinant mNG-ORF68, mNG-ORF68/HA-ORF21, and mNG-ORF68/ORF21.stop BACs were digested with RsrII to assess that no large-scale rearrangements occurred during cloning. **B.** Western blot of whole cell lysate (25 µg) from unreactivated or reactivated WT iSLK cells or iSLK cells harboring the ORF21.stop BAC, representative of 4 biological replicates.

bioRxiv preprint doi: https://doi.org/10.64898/2026.02.23.707506; this version posted February 23, 2026. The copyright holder for this preprint (which was not certified by peer review) is the author/funder, who has granted bioRxiv a license to display the preprint in perpetuity. It is made available under aCC-BY 4.0 International license.

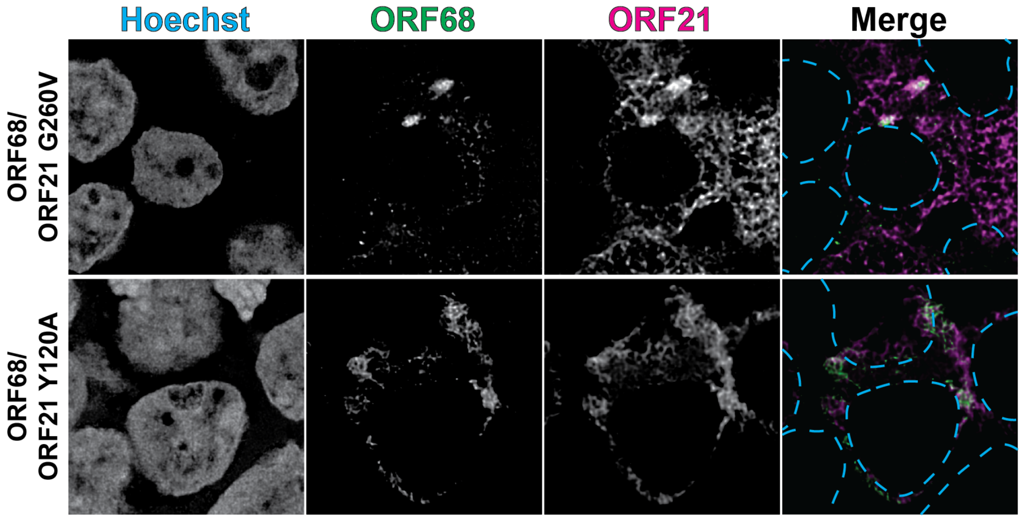

SUPPLEMENTARY FIGURE S5. Mutations in the N-terminus and catalytic domain of ORF21 do not alter the localization of ORF68. Z-slices of transfected HEK293T cells expressing HA-ORF68 and full-length ORF21-strep with mutations G260V and Y120A. Stained for Hoechst (cyan dashed lines), HA-tag (green), and Strep-tag (magenta), representative of at least 5 cells from 1 biological replicate. Scale bars are 10 µm.

bioRxiv preprint doi: https://doi.org/10.64898/2026.02.23.707506; this version posted February 23, 2026. The copyright holder for this preprint (which was not certified by peer review) is the author/funder, who has granted bioRxiv a license to display the preprint in perpetuity. It is made available under aCC-BY 4.0 International license.

SUPPLEMENTARY TABLE 1: TurboID MS dataset listing all cellular and viral proteins identified across all streptavidin enrichments and replicates.

bioRxiv preprint doi: https://doi.org/10.64898/2026.02.23.707506; this version posted February 23, 2026. The copyright holder for this preprint (which was not certified by peer review) is the author/funder, who has granted bioRxiv a license to display the preprint in perpetuity. It is made available under aCC-BY 4.0 International license.

SUPPLEMENTARY TABLE 2: DNA primers used for inverse PCR cloning and qPCR analysis.

**Name**	**Purpose**	**Sequence**
HA-ORF68-F	Cloning	TACCCATACGATGTTCCAGATTACGCTggggcggccatgtttgttccctggcaactc
HA-ORF68-R	Cloning	cattggtaagcttaagtttaaacgctagag
ORF21-1-248-R	Cloning	cacggtcacaggcgttctaatgaggtgcac
ORF21-1-248-STREP-F	Cloning	tcggggcggccgctcgagggaggcggtgga
ORF21-trunc-R	Cloning	catgaattccaccacactggactagtggatccgagc
ORF21-248-580-F	Cloning	gtggactacaggaatgtttatttgctttactta
ORF21-Y120A-F	Cloning	GCCgcgccaatggatcgcttcgccttccagagc
ORF21-Y120-R	Cloning	gtctcccgagtcgtcgtcagtcgctgacaac
ORF21-G260V-F	Cloning	GTGgtaatgggtgtgggcaaatcaacgctggtc
ORF21-G260V-R	Cloning	ctctaagtaaagcaaataaacattcctgta
ORF57Pr-F	qPCR	cagtgttttgccagcaagtg
ORF57Pr-R	qPCR	gggctattttgggaacctg
CTGFPr-F	qPCR	cgaggaatgtccctgtttgt
CTGFPr-R	qPCR	actggctgtctcctctcagc

bioRxiv preprint doi: https://doi.org/10.64898/2026.02.23.707506; this version posted February 23, 2026. The copyright holder for this preprint (which was not certified by peer review) is the author/funder, who has granted bioRxiv a license to display the preprint in perpetuity. It is made available under aCC-BY 4.0 International license.

SUPPLEMENTARY TABLE 3: Double-stranded DNA "geneblocks" used for BAC mutagenesis.

**Name**	**Description of change**	**Full geneblock sequence used for mutagenesis, where bold/underline indicates the mutation**
mIFP	Replace constitutive eGFP reporter with mIFP	gtttttttcttccatttcaggtgtcgtgaggaattagcttggtactaatacgactcactatagggagacccaagctggctaggtaagcttggtaccgagctcggatccactagtccgccaccatg**tcggtaccgctgactacctcagcattcggccacgcgtttctggctaactgtgaacgcgagcagatccacctggcgggctccattcagccgcacggtatcctgctggctgtgaaagagccggacaacgtggtgatccaggcttctattaacgctgcggagttcctgaacaccaactctgttgttggccgtccgctgcgtgacctgggcggcgatctgcctttgcagatcctgccgcacctgaacggcccgctgcacctggctccgatgaccctgcgttgtaccgtgggttctccgccgcgtcgtgtggactgtaccattcatcgtccgtctaacggcggcctgatcgtagaactggaaccagcaaccaagaccactaacattgcgccggctctggacggtgcgtttcatcgtatcacttcttcatcctccctgatgggcctgtgtgacgaaaccgcgactattatccgtgagattactggctacgaccgtgtgatggtagtacgtttcgatgaagagggtaatggcgaaattctgtccgaacgtcgtcgtgcggacctggaagcgttcctgggtaaccgctacccggcgtctactattccgcagatcgctcgtcgcctgtacgaacataaccgtgttcgcctgctggtagatgtgaactatactccggttccgctacagccgcgcatcagcccgctgaacggtcgtgatctggatatgtccctgtcttgcctgcgctctatgtccccgatccaccagaaatacatgcaggacatgggcgttggcgcgaccctggtttgctctctgatggtgtctggtcgtctgtggggtctgatcgcttgccaccactacgaaccgcgcttcgttccgttccacattcgcgctgctggcgaagcgctggcggaaacttgtgcgatccgcatcgcgacgctggagagctttgcacagtctcagtccaaa**tgaagcggccgcgactcggccgcactagaggaattcaggatgacgacgataagtagggataacagggtaatcgatttattcaacaaagccacgttgtgtctcaaaatctctgatgttacattgcacaagataaaaatatatcatcatgaacaataaaactgtctgcttacataaacagtaatacaaggggtgttatgagccatattcaacgggaaacgtcttgctcgaggccgcgattaaattccaacatggatgctgatttatatgggtataaatgggctcgcgataatgtcgggcaatcaggtgcgacaatctatcgattgtatgggaagcccgatgcgccagagttgtttctgaaacatggcaaaggtagcgttgccaatgatgttacagatgagatggtcagactaaactggctgacggaatttatgcctcttccgaccatcaagcattttatccgtactcctgatgatgcatggttactcaccactgcgatccccgggaaaacagcattccaggtattagaagaatatcctgattcaggtgaaaatattgttgatgcgctggcagtgttcctgcgccggttgcattcgattcctgtttgtaattgtccttttaacagcgatcgcgtatttcgtctcgctcaggcgcaatcacgaatgaataacggtttggttgatgcgagtgattttgatgacgagcgtaatggctggcctgttgaacaagtctggaaagaaatgcataagcttttgccattctcaccggattcagtcgtcactcatggtgatttctcacttgataaccttatttttgacgaggggaaattaataggttgtattgatgttggacgagtcggaatcgcagaccgataccaggatcttgccatcctatggaactgcctcggtgagttttctccttcattacagaaacggctttttcaaaaatatggtattgataatcctgatatgaataaattgcagtttcatttgatgctcgatgagtttttctaatcagaattggttaattggttcgagctcggatccactagtccgccaccatg**tcggtaccgctgactacctcagcattcggccacgcgtttctggctaactgtgaacgcgagcagatccacctggcgggctccattcagccgcacggtatcctgctggctgtgaaagagccggacaacgtggtgatccaggcttctattaacgctgcggagttcctgaacaccaactctgttgttggccgtccgctgcgtgacctgggcggcgatctgcctttgcagatcctgccgcacctgaacggcccgctgcacctggctccgatgaccctgcgttgtaccgtgggttctccgccgcgtcgtgtggactgtaccattcatcgtccgtctaacggcggcctgatcgtagaactggaaccagcaaccaagaccactaacattgcgccggctctggacggtgcgtttcatcgtatcacttcttcatcctccctgatgggcctgtgtgacgaaaccgcgactattatccgtgagattactggctacgaccgtgtgatggtagtacgtttcgatgaagagggtaatggcgaaattctgtccgaacgtcgtcgtgcggacctggaagcgttcctgggtaaccgctacccggcgtctactattccgcagatcgctcgtcgcctgtacgaacataaccgtgttcgcctgctggtagatgtgaactatactccggttccgctacagccgcgcatcagcccgctgaacggtcgtgatctggatatgtccctgtcttgcctgcgctctatgtccccgatccaccagaaatacatgcaggacatgggcgttggcgcgaccctggtttgctctctgatggtgtctggtcgtctgtggggtctgatcgcttgccaccactacgaaccgcgcttcgttccgttccacattcgcgctgctggcgaagcgctggcggaaacttgtgcgatccgcatcgcgacgctggagagctttgcacagtctcagtccaaa**tgaagcggccgcgactcggccgcactagaggaattccgcccctctccccccccccctctccctcccccccccctaacgttactggccgaagccgcttggaataaggccggtgtgcgtttgtctatatg
TSP-ORF68	Insert Twin-Strep (TSP) tag at N-terminus of ORF68	attcaaatatttgactccgcagcgagccggtttgcagagtggtcacctgccctgctccacacccacccccgcgtctcttccaactctcaactcacgatccagggaaaccaccgtccagtggccatg**agtgcgtggagtcatcctcaattcgagaaaggtggaggttctggcggtggatcgggaggttcagcgtggagccacccgcagttcgaaaaatccggactggaagttctgttccaggggccc**tttgttccctggcaactcggtacaattaccaggatgacgacgataagtagggataacagggtaatcgatttattcaacaaagccacgttgtgtctcaaaatctctgatgttacattgcacaagataaaaatatatcatcatgaacaataaaactgtctgcttacataaacagtaatacaaggggtgttatgagccatattcaacgggaaacgtcttgctcgaggccgcgattaaattccaacatggatgctgatttatatgggtataaatgggctcgcgataatgtcgggcaatcaggtgcgacaatctatcgattgtatgggaagcccgatgcgccagagttgtttctgaaacatggcaaaggtagcgttgccaatgatgttacagatgagatggtcagactaaactggctgacggaatttatgcctcttccgaccatcaagcattttatccgtactcctgatgatgcatggttactcaccactgcgatccccgggaaaacagcattccaggtattagaagaatatcctgattcaggtgaaaatattgttgatgcgctggcagtgttcctgcgccggttgcattcgattcctgtttgtaattgtccttttaacagcgatcgcgtatttcgtctcgctcaggcgcaatcacgaatgaataacggtttggttgatgcgagtgattttgatgacgagcgtaatggctggcctgttgaacaagtctggaaagaaatgcataagcttttgccattctcaccggattcagtcgtcactcatggtgatttctcacttgataaccttatttttgacgaggggaaattaataggttgtattgatgttggacgagtcggaatcgcagaccgataccaggatcttgccatcctatggaactgcctcggtgagttttctccttcattacagaaacggctttttcaaaaatatggtattgataatcctgatatgaataaattgcagtttcatttgatgctcgatgagtttttctaatcagaattggttaattggttgatccagggaaaccaccgtccagtggccatg**agtgcgtggagtcatcctcaattcgagaaaggtggaggttctggcggtggatcgggaggttcagcgtggagccacccgcagttcgaaaaatccggactggaagttctgttccaggggccc**tttgttccctggcaactcggtacaattacccgtcaccgagatgagctccaaaaactactggcagcctccctgctcccggagcacccggaggagagcctcggtaaccccataatgacacagattcacc
mNG-ORF68	Insert mNeonGreen to ORF68 N-terminus	attcaaatatttgactccgcagcgagccggtttgcagagtggtcacctgccctgctccacacccacccccgcgtctcttccaactctcaactcacgatccagggaaaccaccgtccagtggccatg**gtcagcaaaggcgaagaagacaacatggcatcactgccagcgacacacgaacttcatattttcggctccatcaatggtgtggacttcgatatggttggacagggtactgggaaccctaacgatggatatgaggagttgaacttgaagagtaccaagggcgatcttcagttttcaccttggattctcgtacctcatataggttatggctttcatcaatatctgccctatcctgacggtatgtcacctttccaagcagctatggttgacgggagcggttatcaggttcaccggacaatgcagtttgaagatggagcctctttgaccgtaaattatcggtatacttatgagggcagccatattaaaggcgaggctcaagttaaggggactgggttccccgcggatggtcccgtaatgacaaacagtctgacggctgcagactggtgccgctctaagaagacatatccaaatgacaagacgattattagcactttcaagtggtcatacactacaggaaatggtaaacgctataggagcactgcccggaccacgtatactttcgcgaagccgatggcggcaaattatttgaaaaaccaaccgatgtatgtgttcagaaagacagaactcaagcattctaaaactgaactgaacttcaaagagtggcagaaagcctttactgatgtaatgggcatggacgaactctataagtccggactcagatccggtggaggcggatcgggatccggactggaagttctgttccaggggccc**tttgttccctggcaactcggtacaattaccaggatgacgacgataagtagggataacagggtaatcgatttattcaacaaagccacgttgtgtctcaaaatctctgatgttacattgcacaagataaaaatatatcatcatgaacaataaaactgtctgcttacataaacagtaatacaaggggtgttatgagccatattcaacgggaaacgtcttgctcgaggccgcgattaaattccaacatggatgctgatttatatgggtataaatgggctcgcgataatgtcgggcaatcaggtgcgacaatctatcgattgtatgggaagcccgatgcgccagagttgtttctgaaacatggcaaaggtagcgttgccaatgatgttacagatgagatggtcagactaaactggctgacggaatttatgcctcttccgaccatcaagcattttatccgtactcctgatgatgcatggttactcaccactgcgatccccgggaaaacagcattccaggtattagaagaatatcctgattcaggtgaaaatattgttgatgcgctggcagtgttcctgcgccggttgcattcgattcctgtttgtaattgtccttttaacagcgatcgcgtatttcgtctcgctcaggcgcaatcacgaatgaataacggtttggttgatgcgagtgattttgatgacgagcgtaatggctggcctgttgaacaagtctggaaagaaatgcataagcttttgccattctcaccggattcagtcgtcactcatggtgatttctcacttgataaccttatttttgacgaggggaaattaataggttgtattgatgttggacgagtcggaatcgcagaccgataccaggatcttgccatcctatggaactgcctcggtgagttttctccttcattacagaaacggctttttcaaaaatatggtattgataatcctgatatgaataaattgcagtttcatttgatgctcgatgagtttttctaatcagaattggttaattggttgatccagggaaaccaccgtccagtggccatg**gtcagcaaaggcgaagaagacaacatggcatcactgccagcgacacacgaacttcatattttcggctccatcaatggtgtggacttcgatatggttggacagggtactgggaaccctaacgatggatatgaggagttgaacttgaagagtaccaagggcgatcttcagttttcaccttggattctcgtacctcatataggttatggctttcatcaatatctgccctatcctgacggtatgtcacctttccaagcagctatggttgacgggagcggttatcaggttcaccggacaatgcagtttgaagatggagcctctttgaccgtaaattatcggtatacttatgagggcagccatattaaaggcgaggctcaagttaaggggactgggttccccgcggatggtcccgtaatgacaaacagtctgacggctgcagactggtgccgctctaagaagacatatccaaatgacaagacgattattagcactttcaagtggtcatacactacaggaaatggtaaacgctataggagcactgcccggaccacgtatactttcgcgaagccgatggcggcaaattatttgaaaaaccaaccgatgtatgtgttcagaaagacagaactcaagcattctaaaactgaactgaacttcaaagagtggcagaaagcctttactgatgtaatgggcatggacgaactctataagtccggactcagatccggtggaggcggatcgggatccggactggaagttctgttccaggggccct**ttgttccctggcaactcggtacaattacccgtcaccgagatgagctccaaaaactactggcagcctccctgctcccggagcacccggaggagagcctcggtaaccccataatgacacagattcacc

bioRxiv preprint doi: https://doi.org/10.64898/2026.02.23.707506; this version posted February 23, 2026. The copyright holder for this preprint (which was not certified by peer review) is the author/funder, who has granted bioRxiv a license to display the preprint in perpetuity. It is made available under aCC-BY 4.0 International license.

mNG-T2A-ORF68	Insert T2A sequence between mNeonGreen and ORF68	aactcaagcattctaaaactgaactgaacttcaaagagtggcagaaagcctttactgatgtaatgggcatggacgaactctataagtccggactcagatccggtggaggcggatcgggatccgga**gagggcagaggaagtctgctaacatgcggtgacgtcgaggagaatcctggcccaccggtcgccacc**ctggaagttctgttccaggggccctaggatgacgacgataagtagggataacagggtaatcgatttattcaacaaagccacgttgtgtctcaaaatctctgatgttacattgcacaagataaaaatatatcatcatgaacaataaaactgtctgcttacataaacagtaatacaaggggtgttatgagccatattcaacgggaaacgtcttgctcgaggccgcgattaaattccaacatggatgctgatttatatgggtataaatgggctcgcgataatgtcgggcaatcaggtgcgacaatctatcgattgtatgggaagcccgatgcgccagagttgtttctgaaacatggcaaaggtagcgttgccaatgatgttacagatgagatggtcagactaaactggctgacggaatttatgcctcttccgaccatcaagcattttatccgtactcctgatgatgcatggttactcaccactgcgatccccgggaaaacagcattccaggtattagaagaatatcctgattcaggtgaaaatattgttgatgcgctggcagtgttcctgcgccggttgcattcgattcctgtttgtaattgtccttttaacagcgatcgcgtatttcgtctcgctcaggcgcaatcacgaatgaataacggtttggttgatgcgagtgattttgatgacgagcgtaatggctggcctgttgaacaagtctggaaagaaatgcataagcttttgccattctcaccggattcagtcgtcactcatggtgatttctcacttgataaccttatttttgacgaggggaaattaataggttgtattgatgttggacgagtcggaatcgcagaccgataccaggatcttgccatcctatggaactgcctcggtgagttttctccttcattacagaaacggctttttcaaaaatatggtattgataatcctgatatgaataaattgcagtttcatttgatgctcgatgagtttttctaatcagaattggttaattggttcggtggaggcggatcgggatccgga**gagggcagaggaagtctgctaacatgcggtgacgtcgaggagaatcctggcccaccggtcgccacc**ctggaagttctgttccaggggccctttgttccctggcaactcggtacaattacccgtcaccgagatgagctccaaaaactactggcagcctccctgctcccggagcacccggaggagagcctcgg
29-HA	Insert HA epitope tag at ORF29 C-terminus	gggatggagggcagtcatacagcgccaagcaaaagcacatgtcggacgacttacttgtggcagttgtcatggcccattttatggctaccgatgatagacacatgtacaagcccatatccccacaa**ggaggtggcgggtcatacccatacgatgttccagattacgct**taaaagcacagcgctcaattatctaaggatgacgacgataagtagggataacagggtaatcgatttattcaacaaagccacgttgtgtctcaaaatctctgatgttacattgcacaagataaaaatatatcatcatgaacaataaaactgtctgcttacataaacagtaatacaaggggtgttatgagccatattcaacgggaaacgtcttgctcgaggccgcgattaaattccaacatggatgctgatttatatgggtataaatgggctcgcgataatgtcgggcaatcaggtgcgacaatctatcgattgtatgggaagcccgatgcgccagagttgtttctgaaacatggcaaaggtagcgttgccaatgatgttacagatgagatggtcagactaaactggctgacggaatttatgcctcttccgaccatcaagcattttatccgtactcctgatgatgcatggttactcaccactgcgatccccgggaaaacagcattccaggtattagaagaatatcctgattcaggtgaaaatattgttgatgcgctggcagtgttcctgcgccggttgcattcgattcctgtttgtaattgtccttttaacagcgatcgcgtatttcgtctcgctcaggcgcaatcacgaatgaataacggtttggttgatgcgagtgattttgatgacgagcgtaatggctggcctgttgaacaagtctggaaagaaatgcataagcttttgccattctcaccggattcagtcgtcactcatggtgatttctcacttgataaccttatttttgacgaggggaaattaataggttgtattgatgttggacgagtcggaatcgcagaccgataccaggatcttgccatcctatggaactgcctcggtgagttttctccttcattacagaaacggctttttcaaaaatatggtattgataatcctgatatgaataaattgcagtttcatttgatgctcgatgagtttttctaatcagaattggttaattggttcatgtacaagcccatatccccacaa**ggaggtggcgggtcatacccatacgatgttccagattacgct**taaaagcacagcgctcaattatctacagccccccatagccatccattatagcacacaccccgttctaatctggcatgtatattgtacggtagggctcctgggtagctatgcccaccgtcggcgg
HA-67A	Insert HA epitope tag at ORF67A N-terminus	caggtgaccactctgcaaaccggctcgctgcggagtcaaatatttgaatcaattgtttgctccgctccctatgtgcgccgtcgtgtcttggggcgtgtgttcccctccccgtggaacccaacatg**tacccatacgatgttccagattacgctggaggtggcgggtca**gagtacgcgtctgaccagcttctgcaggatgacgacgataagtagggataacagggtaatcgatttattcaacaaagccacgttgtgtctcaaaatctctgatgttacattgcacaagataaaaatatatcatcatgaacaataaaactgtctgcttacataaacagtaatacaaggggtgttatgagccatattcaacgggaaacgtcttgctcgaggccgcgattaaattccaacatggatgctgatttatatgggtataaatgggctcgcgataatgtcgggcaatcaggtgcgacaatctatcgattgtatgggaagcccgatgcgccagagttgtttctgaaacatggcaaaggtagcgttgccaatgatgttacagatgagatggtcagactaaactggctgacggaatttatgcctcttccgaccatcaagcattttatccgtactcctgatgatgcatggttactcaccactgcgatccccgggaaaacagcattccaggtattagaagaatatcctgattcaggtgaaaatattgttgatgcgctggcagtgttcctgcgccggttgcattcgattcctgtttgtaattgtccttttaacagcgatcgcgtatttcgtctcgctcaggcgcaatcacgaatgaataacggtttggttgatgcgagtgattttgatgacgagcgtaatggctggcctgttgaacaagtctggaaagaaatgcataagcttttgccattctcaccggattcagtcgtcactcatggtgatttctcacttgataaccttatttttgacgaggggaaattaataggttgtattgatgttggacgagtcggaatcgcagaccgataccaggatcttgccatcctatggaactgcctcggtgagttttctccttcattacagaaacggctttttcaaaaatatggtattgataatcctgatatgaataaattgcagtttcatttgatgctcgatgagtttttctaatcagaattggttaattggtttcccctccccgtggaacccaacatg**tacccatacgatgttccagattacgctggaggtggcgggtca**gagtacgcgtctgaccagcttctgcctcgtgacatgcaaattttatttcccacgatctattgtagattgaacgccatcaactactgtcagtatttaaaaacctttctcgtccagcgggcacagcc
65-HA	Insert HA epitope tag at ORF65 C-terminus	ccctgtcctctggtccccattcattgtcgccggcgtcagacattctcacaaccctctcatccacgacggaaacggccgcccccgcggtggccgacgcgaggaaaccccccctctggcaaaaagaaa**tacccatacgatgttccagattacgct**taggcaacgattggacaaccgtggaaggatgacgacgataagtagggataacagggtaatcgatttattcaacaaagccacgttgtgtctcaaaatctctgatgttacattgcacaagataaaaatatatcatcatgaacaataaaactgtctgcttacataaacagtaatacaaggggtgttatgagccatattcaacgggaaacgtcttgctcgaggccgcgattaaattccaacatggatgctgatttatatgggtataaatgggctcgcgataatgtcgggcaatcaggtgcgacaatctatcgattgtatgggaagcccgatgcgccagagttgtttctgaaacatggcaaaggtagcgttgccaatgatgttacagatgagatggtcagactaaactggctgacggaatttatgcctcttccgaccatcaagcattttatccgtactcctgatgatgcatggttactcaccactgcgatccccgggaaaacagcattccaggtattagaagaatatcctgattcaggtgaaaatattgttgatgcgctggcagtgttcctgcgccggttgcattcgattcctgtttgtaattgtccttttaacagcgatcgcgtatttcgtctcgctcaggcgcaatcacgaatgaataacggtttggttgatgcgagtgattttgatgacgagcgtaatggctggcctgttgaacaagtctggaaagaaatgcataagcttttgccattctcaccggattcagtcgtcactcatggtgatttctcacttgataaccttatttttgacgaggggaaattaataggttgtattgatgttggacgagtcggaatcgcagaccgataccaggatcttgccatcctatggaactgcctcggtgagttttctccttcattacagaaacggctttttcaaaaatatggtattgataatcctgatatgaataaattgcagtttcatttgatgctcgatgagtttttctaatcagaattggttaattggttgaaaccccccctctggcaaaaagaaa**tacccatacgatgttccagattacgct**taggcaacgattggacaaccgtggagtcacaagtaccacttctttattctgtcaaccgtctccgccagggacgccgatagggcggccactttttgtttgatgcgtctaataaaactaatcagtgt
TurboID-T2A-68	Insert 3xNLS-tagged TurboID with T2A site between TurboID and N-terminus of ORF68	attcaaatatttgactccgcagcgagccggtttgcagagtggtcacctgccctgctccacacccacccccgcgtctcttccaactctcaactcacgatccagggaaaccaccgtccagtggccatg**ggatcagatccaaaaaagaagagaaaggtagacccgaagaaaaagcgtaaggtcgatcccaaaaagaaacggaaagtgggatcaccggtcgccaccaaagacaatactgtgcctctgaagctgatcgctctcctggctaatggcgagttccatagtggcgaacagctgggagaaaccctgggcatgtccagggccgctatcaacaagcacattcagactctgcgcgactggggcgtggacgtgttcaccgtgcccggaaagggctactctctgcccgagcctatcccgctgctgaacgctaaacagattctgggacagctggacggcgggagcgtggcagtcctgcctgtggtc**aggatgacgacgataagtagggataacagggtaatcgatttattcaacaaagccacgttgtgtctcaaaatctctgatgttacattgcacaagataaaaatatatcatcatgaacaataaaactgtctgcttacataaacagtaatacaaggggtgttatgagccatattcaacgggaaacgtcttgctcgaggccgcgattaaattccaacatggatgctgatttatatgggtataaatgggctcgcgataatgtcgggcaatcaggtgcgacaatctatcgattgtatgggaagcccgatgcgccagagttgtttctgaaacatggcaaaggtagcgttgccaatgatgttacagatgagatggtcagactaaactggctgacggaatttatgcctcttccgaccatcaagcattttatccgtactcctgatgatgcatggttactcaccactgcgatccccgggaaaacagcattccaggtattagaagaatatcctgattcaggtgaaaatattgttgatgcgctggcagtgttcctgcgccggttgcattcgattcctgtttgtaattgtccttttaacagcgatcgcgtatttcgtctcgctcaggcgcaatcacgaatgaataacggtttggttgatgcgagtgattttgatgacgagcgtaatggctggcctgttgaacaagtctggaaagaaatgcataagcttttgccattctcaccggattcagtcgtcactcatggtgatttctcacttgataaccttatttttgacgaggggaaattaataggttgtattgatgttggacgagtcggaatcgcagaccgataccaggatcttgccatcctatggaactgcctcggtgagttttctccttcattacagaaacggctttttcaaaaatatggtattgataatcctgatatgaataaattgcagtttcatttgatgctcgatgagtttttctaatcagaattggttaattggtt**gaacgctaaacagattctgggacagctggacggcgggagcgtggcagtcctgcctgtggtcgactccaccaatcagtacctgctggatcgaatcggcgagctgaagagtggggatgcttgcattgcagaatatcagcaggcagggagaggaagcagagggaggaaatggttctctccttttggagctaacctgtacctgagtatgttttggcgcctgaagcggggaccagcagcaatcggcctgggcccggtcatcggaattgtcatggcagaagcgctgcgaaagctgggagcagacaaggtgcgagtcaaatggcccaatgacctgtatctgcaggatagaaagctggcaggcatcctggtggagctggccggaataacaggcgatgctgcacagatcgtcattggcgccgggattaacgtggctatgaggcgcgtggaggaaagcgtggtcaatcagggctggatcacactgcaggaagcagggattaacctggacaggaatactctggccgctacgctgatccgagagctgcgggcagccctggaactgttcgagcaggaaggcctggctccatatctgccacggtgggagaagctggataacttcatcaatagacccgtgaagctgatcattggggacaaagagattttcgggattagccgggggattgataaacagggagccctgctgctggaacaggacggagttatcaaaccctggatgggcggagaaatcagtctgcggtctgccgaaaagggatcgggagagggcagaggaagtctgctaacatgcggtgacgtcgaggagaatcctggcccaccggtcgccacc**tttgttccctggcaactcggtacaattacccgtcaccgagatgagctccaaaaactactggcagcctccctgctcccggagcacccggaggagagcctcggtaaccccataatgacacagattcacc
TurboID-68	Insert 3xHA-tagged TurboID at N-terminus of ORF68	attcaaatatttgactccgcagcgagccggtttgcagagtggtcacctgccctgctccacacccacccccgcgtctcttccaactctcaactcacgatccagggaaaccaccgtccagtggccatg**tacccgtatgatgttccggattacgctggctatccctacgacgtgcccgactatgccgggtacccctatgacgtcccagactacgcaggggcggccaaagacaatactgtgcctctgaagctgatcgctctcctggctaatggcgagttccatagtggcgaacagctgggagaaaccctgggcatgtccagggccgctatcaacaagcacattcagactctgcgcgactggggcgtggacgtgttcaccgtgcccggaaagggctactctctgcccgagcctatcccgctgctgaacgctaaacagattctgggacagctggacggcgggagcgtggcagtcctgcctgtggtcgactccaccaatcagtacctgctggatcgaatcggcgagctgaagagtggggatgcttgcattgcag**

bioRxiv preprint doi: https://doi.org/10.64898/2026.02.23.707506; this version posted February 23, 2026. The copyright holder for this preprint (which was not certified by peer review) is the author/funder, who has granted bioRxiv a license to display the preprint in perpetuity. It is made available under aCC-BY 4.0 International license.

		**aatatcagcaggcagggagaggaagcagagggaggaaatggttctctccttttggagctaacctgtacctgagtatgttttggcgcctgaagcggggaccagcagcaatcggcctgggcccggtcatcggaattgtcatggcagaagcgctgcgaaagctgggagcagacaaggtgcgagtcaaatggcccaatgacctgtatctgcaggatagaaagctggcaggcatcctggtggagctggccggaataacaggcgatgctgcacagatcgtcattggcgccgggattaacgtggctatgaggcgcgtggaggaaagcgtggtcaatcagggctggatcacactgcaggaagcagggattaacctggacaggaatactctggccgctacgctgatccgagagctgcgggcagccctggaactgttcgagcaggaaggcctggctccatatctgccacggtgggagaagctggataacttcatcaatagacccgtgaagctgatcattggggacaaagagattttcgggattagccgggggattgataaacagggagccctgctgctggaacaggacggagttatcaaaccctggatgggcggagaaatcagtctgcggtctgccgaaaagtccggactggaagttctgttccaggggccc**tttgttccctggcaactcggtacaattaccaggatgacgacgataagtagggataacagggtaatcgatttattcaacaaagccacgttgtgtctcaaaatctctgatgttacattgcacaagataaaaatatatcatcatgaacaataaaactgtctgcttacataaacagtaatacaaggggtgttatgagccatattcaacgggaaacgtcttgctcgaggccgcgattaaattccaacatggatgctgatttatatgggtataaatgggctcgcgataatgtcgggcaatcaggtgcgacaatctatcgattgtatgggaagcccgatgcgccagagttgtttctgaaacatggcaaaggtagcgttgccaatgatgttacagatgagatggtcagactaaactggctgacggaatttatgcctcttccgaccatcaagcattttatccgtactcctgatgatgcatggttactcaccactgcgatccccgggaaaacagcattccaggtattagaagaatatcctgattcaggtgaaaatattgttgatgcgctggcagtgttcctgcgccggttgcattcgattcctgtttgtaattgtccttttaacagcgatcgcgtatttcgtctcgctcaggcgcaatcacgaatgaataacggtttggttgatgcgagtgattttgatgacgagcgtaatggctggcctgttgaacaagtctggaaagaaatgcataagcttttgccattctcaccggattcagtcgtcactcatggtgatttctcacttgataaccttatttttgacgaggggaaattaataggttgtattgatgttggacgagtcggaatcgcagaccgataccaggatcttgccatcctatggaactgcctcggtgagttttctccttcattacagaaacggctttttcaaaaatatggtattgataatcctgatatgaataaattgcagtttcatttgatgctcgatgagtttttctaatcagaattggttaattggttgatccagggaaaccaccgtccagtggccatg**tacccgtatgatgttccggattacgctggctatccctacgacgtgcccgactatgccgggtacccctatgacgtcccagactacgcaggggcggccaaagacaatactgtgcctctgaagctgatcgctctcctggctaatggcgagttccatagtggcgaacagctgggagaaaccctgggcatgtccagggccgctatcaacaagcacattcagactctgcgcgactggggcgtggacgtgttcaccgtgcccggaaagggctactctctgcccgagcctatcccgctgctgaacgctaaacagattctgggacagctggacggcgggagcgtggcagtcctgcctgtggtcgactccaccaatcagtacctgctggatcgaatcggcgagctgaagagtggggatgcttgcattgcagaatatcagcaggcagggagaggaagcagagggaggaaatggttctctccttttggagctaacctgtacctgagtatgttttggcgcctgaagcggggaccagcagcaatcggcctgggcccggtcatcggaattgtcatggcagaagcgctgcgaaagctgggagcagacaaggtgcgagtcaaatggcccaatgacctgtatctgcaggatagaaagctggcaggcatcctggtggagctggccggaataacaggcgatgctgcacagatcgtcattggcgccgggattaacgtggctatgaggcgcgtggaggaaagcgtggtcaatcagggctggatcacactgcaggaagcagggattaacctggacaggaatactctggccgctacgctgatccgagagctgcgggcagccctggaactgttcgagcaggaaggcctggctccatatctgccacggtgggagaagctggataacttcatcaatagacccgtgaagctgatcattggggacaaagagattttcgggattagccgggggattgataaacagggagccctgctgctggaacaggacggagttatcaaaccctggatgggcggagaaatcagtctgcggtctgccgaaaagtccggactggaagttctgttccaggggccc**tttgttccctggcaactcggtacaattacccgtcaccgagatgagctccaaaaactactggcagcctccctgctcccggagcacccggaggagagcctcggtaaccccataatgacacagattcaccag
21-HA	Insert HA epitope tag at C-terminus of ORF21	gcctgtggaccgaaatctacaggcagatcctgtccaatccggctattaaacccagggccatcaactggccagcattagagagccagtctaaagcagttaatcacctagaggagacatgcagggtc**ggaggatacccatacgatgttccagattacgcttag**catgcagggtctagccttcttggcgaggatgacgacgataagtagggataacagggtaatcgatttattcaacaaagccacgttgtgtctcaaaatctctgatgttacattgcacaagataaaaatatatcatcatgaacaataaaactgtctgcttacataaacagtaatacaaggggtgttatgagccatattcaacgggaaacgtcttgctcgaggccgcgattaaattccaacatggatgctgatttatatgggtataaatgggctcgcgataatgtcgggcaatcaggtgcgacaatctatcgattgtatgggaagcccgatgcgccagagttgtttctgaaacatggcaaaggtagcgttgccaatgatgttacagatgagatggtcagactaaactggctgacggaatttatgcctcttccgaccatcaagcattttatccgtactcctgatgatgcatggttactcaccactgcgatccccgggaaaacagcattccaggtattagaagaatatcctgattcaggtgaaaatattgttgatgcgctggcagtgttcctgcgccggttgcattcgattcctgtttgtaattgtccttttaacagcgatcgcgtatttcgtctcgctcaggcgcaatcacgaatgaataacggtttggttgatgcgagtgattttgatgacgagcgtaatggctggcctgttgaacaagtctggaaagaaatgcataagcttttgccattctcaccggattcagtcgtcactcatggtgatttctcacttgataaccttatttttgacgaggggaaattaataggttgtattgatgttggacgagtcggaatcgcagaccgataccaggatcttgccatcctatggaactgcctcggtgagttttctccttcattacagaaacggctttttcaaaaatatggtattgataatcctgatatgaataaattgcagtttcatttgatgctcgatgagtttttctaatcagaattggttaattggtttcacctagaggagacatgcagggtc**ggaggatacccatacgatgttccagattacgcttag**catgcagggtctagccttcttggcggcccttgcatgctggcgatgcatatcgttgacatgtggagccactggcgcgttgccgacaacggcgacgacaataacccgctccgccacgcagctcatcaa
21.stop	Add 2 stop codons in ORF21 coding region	caaccagcacggatatggacgacctccctgaggagaggaaaccactaacgggaaagtctgtaaaaacctcgtacatatacgacgtgcccaccgtcccgaccagcaagccg**tagcattga**atgcacgacaactctctctacgcaacgaggatgacgacgataagtagggataacagggtaatcgatttattcaacaaagccacgttgtgtctcaaaatctctgatgttacattgcacaagataaaaatatatcatcatgaacaataaaactgtctgcttacataaacagtaatacaaggggtgttatgagccatattcaacgggaaacgtcttgctcgaggccgcgattaaattccaacatggatgctgatttatatgggtataaatgggctcgcgataatgtcgggcaatcaggtgcgacaatctatcgattgtatgggaagcccgatgcgccagagttgtttctgaaacatggcaaaggtagcgttgccaatgatgttacagatgagatggtcagactaaactggctgacggaatttatgcctcttccgaccatcaagcattttatccgtactcctgatgatgcatggttactcaccactgcgatccccgggaaaacagcattccaggtattagaagaatatcctgattcaggtgaaaatattgttgatgcgctggcagtgttcctgcgccggttgcattcgattcctgtttgtaattgtccttttaacagcgatcgcgtatttcgtctcgctcaggcgcaatcacgaatgaataacggtttggttgatgcgagtgattttgatgacgagcgtaatggctggcctgttgaacaagtctggaaagaaatgcataagcttttgccattctcaccggattcagtcgtcactcatggtgatttctcacttgataaccttatttttgacgaggggaaattaataggttgtattgatgttggacgagtcggaatcgcagaccgataccaggatcttgccatcctatggaactgcctcggtgagttttctccttcattacagaaacggctttttcaaaaatatggtattgataatcctgatatgaataaattgcagtttcatttgatgctcgatgagtttttctaatcagaattggttaattggttccgtcccgaccagcaagccg**tagcattga**atgcacgacaactctctctacgcaacgcctaggtttccgcccagacctctcatacggcacccttccgaaaaaggcagcatttttgccagtcggttgtcagcgactgacgac

bioRxiv preprint doi: https://doi.org/10.64898/2026.02.23.707506; this version posted February 23, 2026. The copyright holder for this preprint (which was not certified by peer review) is the author/funder, who has granted bioRxiv a license to display the preprint in perpetuity. It is made available under aCC-BY 4.0 International license.

SUPPLEMENTARY TABLE 4: Primary and secondary antibodies used for western blot and immunofluorescence analysis.

Antibody	Manufacturer	Part#	Host	Clonality	Use	Dilution
Vinculin	Abcam	ab91459	rabbit	polyclonal	Western	1:1,000
BirA (mutated TurboID)	Agrisera	AS20 4440	rabbit	polyclonal	Western	1:5,000
ORF6	Didychuk et. al 2020[98]	-	rabbit	polyclonal	Western	1:10,000
ORF26	US Biological	524848	mouse	monoclonal	Western	1:1,000
ORF68	Gardner & Glaunsinger 2018[29]	-	rabbit	polyclonal	Western	1:5,000
K8.1	Didychuk et. al 2020[98]	-	rabbit	polyclonal	Western	1:10,000
HA epitope tag	Cell Signaling	2367S	mouse	monoclonal	Western	1:1,000
Streptavidin-HRP	Invitrogen	S911	-	-	Western	1:500
GAPDH	ThermoFisher	AM4300	mouse	monoclonal	Western	1:5,000
Strep tag II	Invitrogen	PIMA537747	mouse	monoclonal	Western	1:500
Anti-Strep-tag II	Abcam	ab307676	rabbit	monoclonal	Western, IF primary	1:1,000, 1:100
Recombinant Anti-Lamin A + Lamin C	Abcam	ab133256	rabbit	monoclonal	IF primary	1:200
HA-Tag (F-7)	Santa Cruz Biotechnology	sc-7392	mouse	monoclonal	IF primary	1:500
HHV-8 PF-8 (ORF59)	MilliporeSigma	MABF2752	mouse	monoclonal	IF primary	1:200
Nucleolin	Cell Signaling	14574S	rabbit	monoclonal	IF primary	1:1000
G3BP1	Invitrogen	704023	rabbit	polyclonal	IF primary	1:100
PMP70	Invitrogen	PA1-650	rabbit	polyclonal	IF primary	1:200
LAMP1	Invitrogen	MA5-29385	rabbit	monoclonal	IF primary	1:100
Streptavidin, Alexa Fluor™ 568 conjugate	Invitrogen	S11226	-	-	IF secondary	1:200
Alexa Fluor® 680 AffiniPure™ Donkey Anti-Mouse IgG (H+L)	Jackson ImmunoResearch	715-625-150	donkey	polyclonal	IF secondary	1:200
Alexa Fluor® 680 AffiniPure Donkey Anti-Rabbit IgG (H+L)	Jackson ImmunoResearch	711-625-152	donkey	polyclonal	IF secondary	1:200
Alexa Fluor® 594 AffiniPure AffiniPure Donkey Anti-Rabbit IgG (H+L)	Jackson ImmunoResearch	711-585-152	donkey	polyclonal	IF secondary	1:200

bioRxiv preprint doi: https://doi.org/10.64898/2026.02.23.707506; this version posted February 23, 2026. The copyright holder for this preprint (which was not certified by peer review) is the author/funder, who has granted bioRxiv a license to display the preprint in perpetuity. It is made available under aCC-BY 4.0 International license.

Alexa Fluor® 594 AffiniPure Donkey Anti-Mouse IgG (H+L)	Jackson ImmunoResearch	715-585-150	donkey	polyclonal	IF secondary	1:200
Alexa Fluor® 488 AffiniPure Donkey Anti-Mouse IgG (H+L)	Jackson ImmunoResearch	715-545-150	donkey	polyclonal	IF secondary	1:200
Alexa Fluor™ Plus 405 Phalloidin	ThermoFisher	A30104	-	-	IF secondary	1:400
